# Urinary Fractional Excretion of Phosphorus in Dogs with Spontaneous Chronic Kidney Disease

**DOI:** 10.3390/vetsci4040067

**Published:** 2017-12-14

**Authors:** Cínthia Ribas Martorelli, Márcia Mery Kogika, Fernanda Chicharo Chacar, Douglas Segalla Caragelasco, Ana Carolina Brandão de Campos Fonseca Pinto, Carla Aparecida Batista Lorigados, Lúcia Conceição Andrade

**Affiliations:** 1 Department of Veterinary Internal Medicine, School of Veterinary Medicine and Animal Science, University of São Paulo, Av. Prof. Dr. Orlando Marques de Paiva, 87, São Paulo, SP 05508-270, Brazil; mmkogika@usp.br (M.M.K.); fernandachicharo@gmail.com (F.C.C.); mv.douglas@yahoo.com.br (D.S.C.); anacarol@usp.br (A.C.B.C.F.P.); clorigados@usp.br (C.A.B.L.); 2 Division of Nephrology, School of Medicine, University of São Paulo, Av. Dr. Arnaldo, 455, São Paulo, SP 01246-903, Brazil; luciacan@usp.br

**Keywords:** canine, hyperphosphatemia, hyperparathyroidism, phosphaturia

## Abstract

The increase of urinary fractional excretion of phosphorus (uFEP) may indicate phosphorus retention before the onset of hyperphosphatemia in the early stages of chronic kidney disease (CKD). The hypothesis of this study is whether uFEP may increase during the early stage of CKD as a compensatory mechanism to prevent hyperphosphatemia as well as whether hyperphosphatemia in the late stages is associated with increase or decrease in uFEP in dogs with naturally occurring CKD; therefore, the aim of this study was to determine the uFEP in CKD dogs with different stages. Forty-nine CKD dogs were included, and they were divided into stage 1 (serum creatinine < 1.4 mg/dL), stage 2 (serum creatinine 1.5 to 2.0 mg/dL), stage 3 (serum creatinine 2.1 to 5.0 mg/dL) and stage 4 (serum creatinine > 5.0 mg/dL), according to the IRIS staging criteria. The stage 3 was subdivided into stage 3-A (serum creatinine 2.1 to 3.5 mg/dL) and stage 3-B (serum creatinine 3.6 to 5.0 mg/dL). The control group comprised 10 dogs, and uFEP ≤ 40% was considered as normal. A progressive increase in uFEP along the progression of CKD was found. However, similar results of uFEP levels were observed in late CKD, since there were no differences between stages 3 (A, B) and 4. Interestingly, some CKD dogs with stage 4 showed normal or reduced uFEP, besides hyperphosphatemia; conversely, some dogs in early CKD had increased uFEP values and normophosphatemia. Our findings suggest that uFEP may act as a compensatory mechanism to avoid the onset of hyperphosphatemia in early CKD, but not in later stages. uFEP assessment may be considered as an additional tool for the diagnostic and monitoring of phosphate disorders in dogs with CKD, since it may help to identify disturbances of phosphorus balance. More studies are needed to elucidate the role of uFEP in phosphorus homeostasis in dogs with CKD.

## 1. Introduction

The kidney plays an essential role in phosphorus homeostasis, since phosphate is primarily excreted in urine. In chronic kidney disease (CKD), the loss of renal mass leads to a decrease in the glomerular filtration rate (GFR), thus reducing the urinary excretion of phosphorus. Phosphate retention, and further hyperphosphatemia, is associated with the development of renal hyperparathyroidism and disease progression. Therefore, the phosphorus control in CKD is a very important therapeutic strategy in order to avoid CKD progression [1,2].

The assessment of serum phosphorus concentrations is routinely used to monitor the phosphate behavior in dogs with CKD. The International Renal Interest Society (IRIS) recommends maintaining serum phosphorus concentrations below 4.6 mg/dL in CKD dogs with stage 2, 5.0 mg/dL in CKD dogs with stage 3 and below 6.0 mg/dL in those CKD dogs with stage 4 [1,2].

However extensively used in the clinical routine, the assessment of serum phosphorus concentration may reflect only late disturbances in phosphate homeostasis. Previous reports have shown that FGF-23 is an early marker of mineral disorder in chronic kidney disease, which has a phosphaturic effect (thus it promotes an increase of uFEP values) in order to avoid the development of hyperphosphatemia, which contributes to the progression of CKD [3,4].

The use of biomarkers such as urinary fractional excretion of phosphorus (uFEP) and serum FGF-23 in face of normal serum phosphorus levels could give early information about the kinetics of phosphorus, and add important information for the better understanding of the pathophysiology [3,4].

Fibroblast growth factor 23 (FGF-23) is a phosphaturic hormone that increases renal excretion of phosphorus in the early stages of CKD, as well as decreases its intestinal absorption via diminished calcitriol [3,4].

Urinary fractional excretion of electrolytes is the fraction of filtered electrolytes that was not reabsorbed and, consequently, was excreted in urine [5,6,7,8,9,10]. In healthy dogs, the uFEP under 40% has been recognized as normal [5,8,11,12], but there are limited investigations in dogs with CKD and IRIS classification was not considered and the study was conducted in a small number of dogs [11,13].

The aim of this study was to determine the uFEP values in CKD dogs with different stages, and the hypothesis is whether uFEP may increase during the early stage of CKD as a compensatory mechanism to prevent hyperphosphatemia as well as whether in the late stages hyperphosphatemia is associated with decrease in uFEP, just as it occurs in humans with CKD.

## 2. Materials and Methods

The study was conducted at Veterinary Teaching Hospital of School of Veterinary Medicine and Animal Science, University of Sao Paulo (Ethical Committee protocol #5436131114). The diagnosis of CKD was based on renal abnormalities detected by abdominal ultrasound, polidipsia and poliuria, weight loss, persistent azotemia (serum creatinine concentration > 1.4 mg/dL) and urine specific gravity below 1.030, in accordance with the International Renal Interest Society criteria for the diagnostic of CKD in dogs. The exclusion criteria were the use of diuretics, glucocorticoids and angiotensin-converting enzyme inhibitor, which may change the uFEP [8]. Hematuria was also an exclusion criterion as blood may affect results of phosphorus measurements [8].

Forty-nine owned dogs with CKD were categorized in accordance with serum creatinine concentration: stage 1 (*n* = 10; serum creatinine < 1.4 mg/dL); stage 2 (*n* = 10; serum creatinine 1.5 to 2.0 mg/dL); stage 3 was subdivided into to stage 3-A (*n* = 9, serum creatinine 2.1 to 3.5 mg/dL) and stage 3-B (*n* = 10, serum creatinine 3.6 to 5.0 mg/dL); and stage 4 (*n* = 10; serum creatinine > 5.0 mg/dL). We have adapted the IRIS staging system [12] particularly for those dogs with stage 3 CKD, because of the wide range of serum creatinine (2.1–5.0 mg/dL) that is normally assumed to classify dogs into that stage. It is expected that dogs with higher serum creatinine concentration (e.g., 5 mg/dL) have more severe loss of renal mass/function than those with lower levels (e.g., 2.1 mg/dL), and then probably greater impairment of renal phosphorus excretion causing hyperphosphatemia as well as marked increase in uFEP. In addition, IRIS recommends CKD classification also based on serum SDMA as only serum creatinine could subestimate the stage; this means dogs with creatinine concentration of 5.0 mg/dL may have SDMA > 45µg/dL that is classified as stage 4 (IRIS, 2015) [12]. If we had only considered stage 3, a large variation of uFEP and serum phosphorus concentrations could be observed and it could cause misinterpretations regarding uFEP results in CKD dogs with stage 3. The subdivision into stage 3-A and stage 3-B was similar to a previous study conducted in CKD cats [14] that also adapted the IRIS staging system for stage 2.

During blood sampling, CKD dogs as well as the clinically normal dogs were not dehydrated, and urine culture was negative in all animals. Control dogs were 5.1 ± 2.5 (mean ± SD) year-old, six females and four males, and composed of mixed-breed (*n* = 5), Pinscher (*n* = 2), Golden Retriever (*n* = 1) and Labrador Retriever (*n* = 1).

In CKD dogs (*n* = 49), the mean ± SD of age in stage 1 was 7.7 ± 2.9 years. CKD dogs were composed of different breeds (14.3% Mixed-breed, 10.2% Yorkshire, 8.2% Teckel, 8.2% Boxer, 8.2% Golden Retriever, 6.1% Labrador Retriever, 6.1% Poodle, 6.1% Schnauzer miniature, 4.1% Pug, 4.1% Maltese, 4.1% American Bulldog, 4.1% Pinscher, 4.1% Brazilian Terrier, 4.1% Pit bull, 2.0% Bull terrier, 2.0% Rotweiller, 2.0% Weimaraner and 2.0% Lhasa Apso; 48.9% female and 51.1% male, aging from 1 to 17 years-old.

All CKD dogs were normotensive and hydrated. Proteinuria was not determined in this study. All CKD dogs were normotensive. All control dogs were fed commercial maintenance diets, and Table 1 shows the type of diets that CKD dogs were fed in each stage; CKD dogs were fed with homemade food, maintenance commercial food or renal prescription diet (named as kidney diet). The CKD dogs were considered hyperphosphatemics according to target values proposed by IRIS (sP < 4.6 mg/dL for stage 2; <5.0 mg/dL for stage 3; and <6.0 mg/dL for stage 4) [1,2,12].

Control group was composed of 10 clinically healthy dogs, recruited based on normal findings of systolic arterial pressure, fasting glucose, complete blood cell count and biochemical profile (urea, creatinine, phosphorus, total calcium, albumin, total protein, cholesterol, triglycerides, alkaline phosphatase, and alanine transaminase), as well as normal abdominal ultrasound, urinalysis and the USG > 1.040.

Blood and urine samples were obtained after 12-h fasting period at least, during the same visit [6,7,10]. Urinary fractional excretion of phosphorus (uFEP) was determined using a spot urine sample. Urine was aseptically collected by cystocentesis or urethral catheterization for urinalysis (dipstick analysis, USG and sediment analysis) and bacterial culture. After urine centrifugation (10 min at 1000 rpm), the supernatant was stored at −80 °C. The serum and urinary inorganic phosphorus was determined using commercial kit (Phosphorus-Fosfomolibdato/UV, cod. 11508, Biosystems, Barcelona, Spain). The uFEP was calculated according to the formula described below [6,7,8,9,10].

$$uFEP\;\left(\%\right)=\;\frac{\left(urinary\;phosphorus\right)/\left(serum\;phosphorus\right)}{\left(urinary\;creatinine\right)/\left(serum\;creatinine\right)}\;\times \;100$$

### Statistical Analysis

Statistical analysis was run in Prism (GraphPad©, GraphPad Software, Inc., La Jolla, CA, USA) and Instat softwares (GraphPad Software, Inc., La Jolla, CA, USA). D’Agostino-Pearson omnibus test was performed to evaluate the normality of the data. In addition, the ANOVA-one way and Tukey multiple comparisons tests were used to investigate differences of uFEP and serum phosphorus in different stages of CKD as well as to compare with the control group. Kruskal-Wallis (ANOVA) and Dunn’s multiple comparisons tests were also used for serum creatinine and urea evaluation. Spearman rank correlation test was performed to evaluate the correlation between serum creatinine versus serum phosphorus, uFEP versus serum creatinine, and uFEP versus serum phosphorus. Statistical significance was set at *p* < 0.05. Graphs of mean and standard deviation and box plot were also performed.

## 3. Results

**Figure 1:**
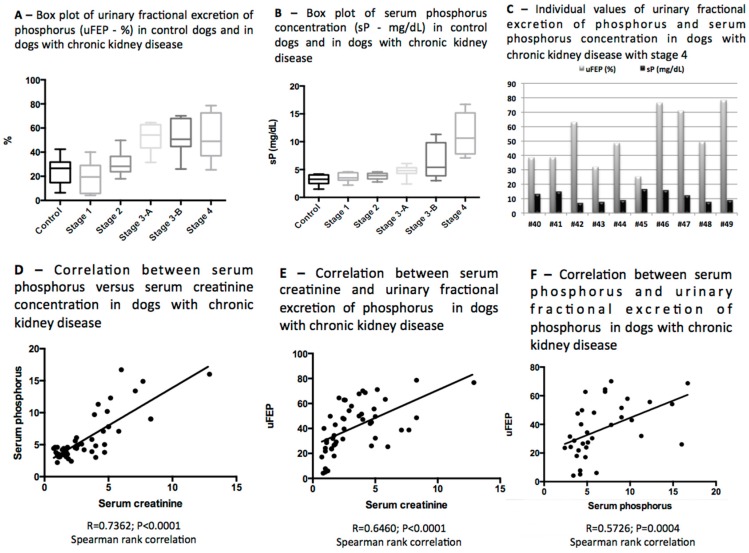
(**A**) Box plot of urinary fractional excretion of phosphorus (uFEP; %); (**B**) Box plot of serum phosphorus concentration (mg/dL); (**C**) individual values of urinary fractional excretion of phosphorus (%) and serum phosphorus concentration (mg/dL) in dogs with chronic kidney disease with stage 4; (**D**) correlation between serum phosphorus (mg/dL) and serum creatinine concentration (mg/dL) in dogs with chronic kidney disease; (**E**) correlation between serum creatinine (mg/dL) and urinary fractional excretion of phosphorus (%) in dogs with chronic kidney disease; (**F**) correlation between serum phosphorus (mg/dL) and urinary fractional excretion of phosphorus (%) in dogs with chronic kidney disease.

**Figure 2:**
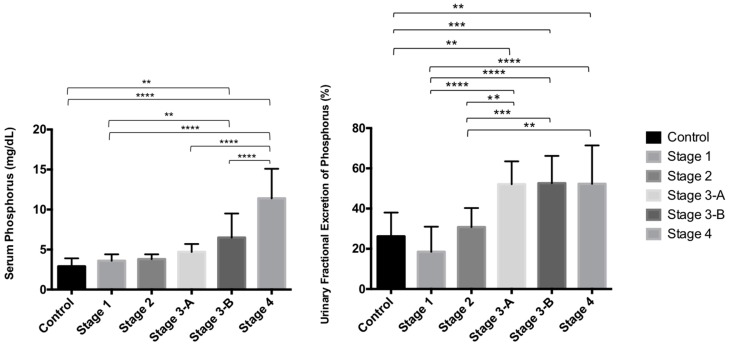
Data in graph are presented as mean ± standard deviation (SD) and statistical analysis (*p* values—Turkey multiple comparison test) of serum phosphorus concentration (sP; mg/dL) and urinary fractional excretion of phosphorus (uFEP; %) in control dogs (C) and in dogs with chronic kidney disease with stage 1 (St 1), stage 2 (St 2), stage 3-A (St 3-A), stage 3-B (St 3-B) and stage 4 (St 4). ** *p* < 0.01; *** *p* < 0.001; **** *p* < 0.0001.

The mean of serum phosphorus concentrations (sP) was markedly increased in CKD dogs with stage 3-B and stage 4, and the mean of uFEP was increased in CKD dogs from stages 3-A to 4 (uFEP > 52%) (Table 2). There was a significant difference in sP of CKD dogs with stage 4 in comparison with control and other stages. There were also differences of uFEP values in CKD dogs with stage 4 in comparison with control and stages 1 and 2; however, no significant differences were observed in CKD dogs with stages 3-A and 3-B in comparison with CKD dogs with stage 4 (Table 2; Figure 1 and Figure 2).

As an overview, increased serum concentration of phosphorus was detected in 18 out of 49 of all CKD dogs (36.7% of all CKD dogs), and taken as target values for sP proposed by IRIS according to the stage of CKD, hyperphosphatemia was observed 8/19 CKD dogs with stage 3 (sP > 5.0 mg/dL) and all CKD dogs with stage 4 had hyperphosphatemia (sP > 6.0 mg/dL). Positive correlation between serum phosphorus and serum creatinine was noticed among all CKD dogs (r = 0.7362; *p* < 0.0001—Spearman rank correlation) (Table 2; Figure 1D and Figure 2).

Gradual increase in uFEP was detected as the CKD progressed, however, in stage 4 the mean percentage of uFEP was similar to the stages 3-A and 3-B (Table 2; Figure 1 and Figure 2), and those values did not increase proportionally to sP concentrations as expected.

In the individual evaluation, none of CKD dogs with stage 1 had increase in uFEP or in sP, in CKD dogs with stage 2, 2/10 that had increase in uFEP were normophosphatemic. In stage 3-A, 2/9 dogs that had increase in uFEP also had increase in serum phosphorus concentrations, and 5/9 that showed increase in uFEP had serum phosphorus concentrations less than 5 mg/dL; in the stage 3-B, 4/10 dogs that had increased uFEP were also hyperphosphatemic (sP > 5 mg/dL), and 3/10 that had sP < 5 mg/dL showed increase in uFEP. In the stage 4, the increase in uFEP was noticed in 6 out of 10 CKD dogs that also were hyperphosphatemic (sP > 6 mg/dL), and in the remaining 4/10 hyperphosphatemic dogs (# 41, #42, #44, #46), the uFEP was decreased (uFEP < 40%) (Figure 1C).

Positive moderate correlation between serum creatinine and uFEP in all CKD dogs was detected (r = 0.6460; *p* < 0.0001—Spearman rank correlation; Figure 1E), also positive moderate correlation between uFEP and serum phosphorus concentration was observed in all CKD dogs (r = 0.5726; *p* = 0.0004—Spearman rank correlation; Figure 1F).

In relation to the diet, the only CKD dog with stage 1 that was fed commercial renal diet showed the lowest value of uFEP (uFEP = 4.2%) and normophosphatemia. In CKD dogs with stage 2, 4/10 were fed renal diet and they had mean of serum phosphorus concentration of 3.5 mg/dL and uFEP of 33.5%, and the remaining dogs in that stage (6/10) were fed homemade food and had mean of 3.7 mg/dL of serum phosphorus concentration and 28.9% of uFEP. All dogs of stage 3-A were fed renal diet and had mean of serum phosphorus concentration and uFEP of 4.7 mg/dL and 52.1%, respectively. In the stage 3-B, 7 out of 9 were fed renal diet and showed the mean of serum phosphorus concentration and uFEP of 6.5 mg/dL and 52.6%, respectively, and 2 out of 9 were fed maintenance homemade diet and had the mean of serum phosphorus concentration and uFEP of 10.5 mg/dL and 60.1%, respectively. In stage 4, 7/10 were fed renal diet and had the mean of serum phosphorus concentration and uFEP of 13.3 mg/dL and 37.9%, respectively, the remaining dogs of stage 4 were fed homemade food and maintenance diet, and showed serum phosphorus concentration and uFEP of 10.7 mg/dL and 58.5%, respectively. Data regarding uFEP and sP among CKD stages in accordance with diet are described in Table 2.

In relation to phosphate-binding agents, the CKD dogs that were treated with aluminum hydroxide were six dogs with stage 3-B (dog #30, #31, #32, #33, #35 and #36) and four CKD dogs with stage 4 (dog #40, #46 and #49). However, dog #21 (stage 3-A) showed hyperphosphatemia (sP = 5.6 mg/dL), and was prescribed aluminum hydroxide as phosphate-binding agent, but in another visit the owners did not follow the therapeutic recommendation, because the dog had constipation as a side effect. Dog #23 (stage 3-A) and #39 (stage 3-B) had hyperphosphatemia (sP = 6.1 mg/dL and 5.8 mg/dL, respectively), and the phosphate binding agent was prescribed, but the owners did not agree to the use of the medication, and the owners did not follow the recommendation.

## 4. Discussion

In our study, a progressive increase was noted in uFEP values among CKD stages, however, in the later stages uFEP levels were similar between CKD dogs with stages 3 (A, B) and 4. Interestingly, in later stages, some dogs showed normal or reduced uFEP despite hyperphosphatemia; on the other hand, in early stages of CKD, some dogs showed increased uFEP despite normophosphatemia. This finding may suggest the role of uFEP as a compensatory mechanism to prevent the onset of hyperphosphatemia in early CKD; in later stages, however, it is likely that this mechanism is not enough, probably because of a marked reduction in renal mass. In addition, it is not yet known about the renal content of Klotho in CKD dogs, and the co-receptor is required for FGF23 action. In humans with CKD, progressive decreases in klotho occurs in the course of CKD secondary to renal resistance to FGF-23 that results in decreased uFEP values and severe hyperphosphatemia [3,4].

Investigations into whether uFEP could be a compensatory mechanism in order to control serum phosphorus levels to avoid overt hyperphosphatemia were conducted in experimental models in dogs [15] as well as in azotemic dogs that were not staged according to IRIS classification [12], and in another study with a few number of dogs with naturally occurring CKD [5]. Our study was conducted in CKD dogs that were classified according to IRIS, and as in the stage 3 there is a wide range of serum creatinine concentrations (from 2.1 to 5.0 mg/dL) and based on a previous study performed by Jepson et al. (2011) [14] in CKD cats, that adapted IRIS staging system in order to avoid in the study group the severe renal dysfunction when serum creatinine concentrations were close to the upper limit (5 mg/dL), the classification as stage 3-A and stage 3-B could allow better understanding for the evaluation of renal phosphorus excretion associated with serum phosphorus levels. In addition, we considered the actual recommendation for IRIS classification that shows that the new biomarker for glomerular filtration rate, symmetric dimethylarginine (SDMA), could detect advanced stage while serum creatinine values could subestimate the CKD stage; this means that dogs with creatinine concentration of 5.0 mg/dL may have SDMA > 45 µg/dL and then it is classified as stage 4 (IRIS, 2015) [12].

Only one CKD dog with stage 1 that was fed with kidney diet showed the lowest values of uFEP and the diet probably contributed to this finding as it is in accordance to Chew et al. (2011) [16]. The hypophosphoric diet may decrease the uFEP as phosphatemia is controlled and also in the early stages, the functional renal mass is still preserved. In the CKD dogs with stage 3-B and stage 4 that were fed with kidney diet had lower concentrations of serum phosphorus than those who were fed with homemade food. This finding could show the importance of kidney diet in the management of CKD dogs in order to control serum phosphorus levels [1,2]. In addition, Block et al., 2004 [17] reported the lowest uFEP values in human patients with CKD when they were on the kidney diet.

The assessment of serum phosphorous is an important tool to investigate the impairment of renal excretion of phosphorus in dogs with CKD [2,18,19,20]. In the present study, hyperphosphatemia was detected in 36.7% of all CKD dogs and it was similar to a previous study in which hyperphosphatemia was observed in 44.2% dogs with CKD secondary to leishmaniasis [21]. In another study, hyperphosphatemia was detected in 68.5% of dogs with CKD, and the prevalence of hyperphosphatemia increased along the progression of CKD: 18% in the stage 1, 40% in stage 2, 92% in the stage 3, and 100% in the stage 4 [18].

In a previous study of uFEP in a fewer number of CKD dogs, the increase in uFEP was described in the late stages [13] as phosphorus retention increases. In our study, similar uFEP levels were noticed in CKD dogs with stages 3-A, 3-B and 4, and mainly in stages 3-B and 4, dogs with hyperphosphatemia did not show an increase in uFEP as a compensatory mechanism was expected. However, the kidneys may lose their efficiency in the later stages of the disease as it has been also observed in human patients with CKD in advanced stages [3,4]. We observed that uFEP had wide variation within stages. Lower uFEP was found in CKD dogs with stage 1 than those dogs in advanced stages, and decrease in uFEP observed in stages 3-A, 3-B and 4 may suggest that the phosphaturic mechanism was not sufficient to maintain normophosphatemia [3,4,22], probably due to the greater loss of nephrons as it has been described in humans as well as in dogs with CKD [1,3,4,16,17,20]. Therefore, the low uFEP associated with hyperphosphatemia is a good indicator to detect the severity of renal dysfunction [3,4,22].

Hyperphosphatemic CKD dogs with stages 3-A, 3-B and 4 that also had an increase in uFEP may indicate that compensatory mechanisms were activated to avoid the development of hyperphosphatemia, and probably FGF-23 and PTH were acting to promote phosphaturia [23]; however in this current study, those biomarkers were not investigated [3,4]. In a previous study in CKD dogs, high values of PTH and FGF-23 were observed [20]. In addition, in a study in CKD human patients with lower values of uFEP, they had higher concentrations of serum FGF-23; this could predict the greater risk of mortality and cardiovascular events [3,4,22,24,25].

Most of the CKD dogs with stages 1 and 2 showed serum phosphorus concentrations within the range according to IRIS recommendations [1,12], and the maintenance of phosphorus serum levels could be a consequence of the FGF-23 mechanism [3,4,23], and FGF-23 could be already released when a slight increase in serum phosphorus developed; this trigger could still happen when serum phosphorus concentrations have slight increases but still within the range considered adequate or normal [3,4,23]. In our study, five CKD dogs with stage 3-A and four CKD dogs with 3-B showed an increase in uFEP values and normophosphatemia. This finding may indicate that the uFEP may be used as an additional biomarker to identify early disturbances of phosphorus homeostasis in normophosphatemic dogs with CKD as a routine procedure, because the measurements of FGF-23 nowadays is still only for research purposes. Thus, this additional information regarding to uFEP associated with serum phosphorus could contribute to detect mechanisms of phosphorus control.

Control of phosphorus levels is an important goal in the management of dogs with chronic kidney disease, since hyperphosphatemia contributes to progression of CKD [1,2,12,16,18,23]. The control may be performed with dietary phosphorus restriction (named as kidney diet) [1,2,12]. Failure to achieve the target serum phosphorus concentration after 4–8 weeks with kidney diet indicates that addition of an intestinal phosphate-binding agent should be considered [1,2,12]. The CKD dogs that received phosphate binding agent were the dog with CKD stage 3-A: dog #30, #31, #32, #33, #35 and #36. The CKD dogs in stage 4 that received phosphate binding agent were dogs #40, #44, #46 and #49. The explanation for other CKD hyperphosphatemic dogs that were not received phosphate-binding agent is described below. Dog #21 presented hyperphosphatemia (sP = 5.6 mg/dL), but did not receive the phosphate-binding agent at the visit. In another situation, when this dog received phosphate binding agent, constipation was noted secondary to the use of aluminum hydroxide. In addition, another phosphate binding agent was proposed, but the owner became resistant to use of this type of therapy even with the knowledge that the hyperphosphatemia may progress the chronic kidney disease. Dogs #23 and #39 presented hyperphosphatemia (sP = 6.1 mg/dL and 5.8 mg/dL, respectively), and in the moment of visit the phosphate-binding agent was prescribed, but the owners did not follow the recommendation.

The limitations of the study were that the control group was not age-matched, and that there were a small number of CKD dogs studied. Thus, these results must be evaluated with caution and additional studies including larger numbers of dogs in different stages of CKD are indicated. Additional limitations are the lack of homogeneous type of diet fed to the control and CKD dogs, and the fact that no fibroblast growth factor 23 (FGF-23) was performed in order to evaluate the phosphaturic action of FGF-23 and its correlation with uFEP. The phosphorus metabolism had a complex physiology with many determining factors and different aspects must be elucidated, and taking into consideration that the evaluation of mineral metabolism would be underestimated only through the serum determination of phosphorus and uFEP, an important limitation of this study was the lack of FGF-23, PTH and vitamin D metabolite measurements.

## 5. Conclusions

Urinary fractional excretion of phosphorus (uFEP) findings may indicate the presence of phosphorus disturbances in CKD dogs, and in advanced stages, the uFEP may help to predict the prognosis as hyperphosphatemia may cause increase in uFEP and the regulation of serum phosphorus levels. A decrease in uFEP associated with hyperphosphatemia may indicate loss of a greater number of nephrons. More investigations, including a prospective evaluation of uFEP as well as serum FGF-23, PTH and vitamin D metabolite measurements, are needed to better understand phosphorus metabolism in dogs with CKD.

## Figures and Tables

**Table 1:** Type of diet (commercial kidney diet—KD, maintenance diet—MT, homemade food—HF), urinary fractional excretion of phosphorus (%; min–max) and serum phosphorus concentrations (sP: mg/dL; min–max) among CKD dogs within different stages (stage 1 to stage 4).

Type of Diet, sP (mg/dL) and uFEP(%)	CKD Stages				
	Stage 1	Stage 2	Stage 3-A	Stage 3-B	Stage 4
**Diet**	KD (*n* = 1) MT (*n* = 6) HF (*n* = 3)	KD (*n* = 4) MT (*n* = 3) HF *n* = 3)	KD (*n* = 10)	KD (*n* = 7) HF (*n* = 2)	KD (*n* = 7) MT (*n* = 2) HF (*n* = 2)
**sP (mg/dL) (min–max)**	2.2–4.6	2.2–4.6	2.4–6.1	3.0–11.3	7.1–16.7
**uFEP (%) (min–max)**	4.2–40.0	17.9–49.8	31.5–64.4	26.1–70.1	25.4–78.6

sP = serum phosphorus concentrations; uFEP = urinary fractional excretion of phosphorus; KD = commercial kidney diet; MT = commercial maintenance diet; HF = homemade food.

**Table 2:** Mean, standard deviation (SD), minimum (min.) and maximum (max.) of serum concentrations of phosphorus (sP; mg/dL) and urinary fractional excretion of phosphorus (uFEP; %) in control dogs and in dogs with chronic kidney disease (Stages 1, 2, 3-A, 3-B and 4).

Control (*n* = 10) Mean ± SD (min.; max.)	2.9 ± 1.0 (1.5–4.2)	26.2 ± 11.8 (6.4–42.4)
Stage 1 (*n* = 10)	3.6 ± 0.8	18.5 ± 12.5
Mean ± SD (min.; max.)	(2.2–4.6)	(4.2–40.0)
Stage 2 (*n* = 10)	3.8 ± 0.6	30.8 ± 9.5
Mean ± SD (min.; max.)	(2.2–4.6)	(17.9–49.8)
Stage 3-A (*n* = 9)	4.7 ± 1.0	52.1 ± 11.4
Mean ± SD (min.; max.)	(2.4–6.1)	(31.5–64.4)
Stage 3-B (*n* = 10)	6.5 ± 3.0	52.6 ± 13.6
Mean ± SD (min.; max.)	(3.0–11.3)	(26.1–70.1)
Stage 4 (*n* = 10)	11.4 ± 3.7	52.3 ± 19.1
Mean ± SD (min.; max.)	(7.1–16.7)	(25.4–78.6)
